# Neural Correlates of Sexual Orientation in Heterosexual, Bisexual, and Homosexual Women

**DOI:** 10.1038/s41598-017-18372-0

**Published:** 2018-01-12

**Authors:** Adam Safron, Victoria Klimaj, David Sylva, A. M. Rosenthal, Meng Li, Martin Walter, J. Michael Bailey

**Affiliations:** 1 Department of Psychology, Northwestern University, Evanston, Illinois USA; 2 Department of Psychiatry, Kaiser Permanente, Oakland, California USA; 3 Department of Psychiatry, Otto von Guericke University, Magdeburg, Germany; 4 Leibniz Institute for Neurobiology, Magdeburg, Germany; 5 Department of Psychiatry, Eberhard Karls University, Tubingen, Germany

## Abstract

We used fMRI to investigate neural correlates of responses to erotic pictures and videos in heterosexual (N = 26), bisexual (N = 26), and homosexual (N = 24) women, ages 25–50. We focused on the ventral striatum, an area of the brain associated with desire, extending previous findings from the sexual psychophysiology literature in which homosexual women had greater category specificity (relative to heterosexual and bisexual women) in their responses to male and female erotic stimuli. We found that homosexual women’s subjective and neural responses reflected greater bias towards female stimuli, compared with bisexual and heterosexual women, whose responses did not significantly differ. These patterns were also suggested by whole brain analyses, with homosexual women showing category-specific activations of greater extents in visual and auditory processing areas. Bisexual women tended to show more mixed patterns, with activations more responsive to female stimuli in sensory processing areas, and activations more responsive to male stimuli in areas associated with social cognition.

## Introduction

Studies using physiological measures have found that women tend to have non-specific patterns of genital arousal^1–3^. That is, in contrast to men, women tend to show similar degrees of arousal to erotic stimuli depicting either sex. For example, heterosexual women have generally shown equivalent arousal to both erotic stimuli featuring men and erotic stimuli featuring women. This has been repeatedly demonstrated with vaginal photoplethysmography^3,4^. This pattern has also been found using less direct measures such as looking time^5^, pupil dilation^6^, and fMRI^7^. Notably, homosexual women’s arousal patterns are more category-specific than heterosexual women’s, although less so than men’s^8^.

The fact that women’s sexual arousal patterns are less category-specific than men’s has been interpreted as a potential contributor to gender differences in “erotic plasticity”^9^, which Baumeister has defined as “the extent to which sex drive is shaped by social, cultural, and situational factors.”

Baumeister offered three lines of evidence when he initially proposed that women may have greater erotic plasticity compared with men: (1) women show larger effects of social and cultural factors on sexual attitudes, desire, and behavior; (2) sexual attitude-behavior consistency is lower in women than in men; (3) individual women exhibit more variation in sexual behavior across time than men. Women’s less specific arousal patterns may also contribute to their increased “sexual fluidity”^10^, which Diamond has defined as an individual’s “capacity for situation-dependent flexibility in sexual responsiveness, which allows individuals to experience changes in same-sex or other-sex desire across both short-term and long-term time periods”^11,12^.

One might hypothesize that arousal patterns of bisexual women should be similar to the non-specific arousal patterns of heterosexual women; however, studies of women’s arousal patterns have mostly neglected to include bisexual women. Heterosexual women’s arousal does not appear to favor erotic stimuli of either sex, and thus may be considered to reflect a bisexual pattern. (We do not mean to imply that heterosexual women are confused or in denial about their “real preferences”; rather, the findings in need of explanation are why heterosexual women show non-heterosexual arousal patterns in the laboratory). The implication of women’s non-specific arousal patterns for their sexual orientations is difficult to interpret. Most women, like most men, behave and identify heterosexually^13–18^. However, men are more likely than women to identify as completely heterosexual or completely homosexual, and women are more likely than men to identify as bisexual or “mostly heterosexual”^19^.

If arousal patterns are similar between heterosexual and bisexual women, the question remains what distinguishes the two groups. One possibility, supported by some research, is that bisexual women tend to have greater sexual motivation, which may increase the likelihood of exploring a capacity for attraction to both sexes^20,21^. Or, bisexual women may be more aware than heterosexual women of their non-specific arousal^22^, which could partially contribute to bisexual sexual motivation. Alternatively, bisexual women may be more likely than heterosexual women to interpret their non-specific arousal states in sexual or romantic terms.

It is also possible that bisexual women’s arousal patterns differ from those observed in heterosexual women. Consistent with this possibility, recent studies suggest that women with bisexual interests tend to be more aroused by female than by male erotic stimuli^23–25^. Perhaps for some women with female-biased arousal patterns, this bias can motivate non-heterosexual feelings, behavior, and identity.

Interpretations of non-specific arousal patterns in women are further complicated by the fact that female genital arousal exhibits relatively low correlations with subjectively reported sexual arousal, in contrast to the high correlations observed in men^26^. Discrepancies between existing genital and subjective measures indicate that some women may report substantial subjective arousal without substantial genital arousal, and vice versa. It has also been suggested that non-specific arousal patterns may not indicate affective responses to erotic stimuli, but may instead reflect a kind of protective preparatory response^27^.

Neuroimaging assessments may shed light on the neural systems that are involved in responding to a given paradigm. Functional magnetic resonance imaging (fMRI) is a neuroimaging approach that allows for the indirect assessment of brain activity by tracking ratios of oxygenated and deoxygenated blood a proxy for neural firing. When used in the context of presenting erotic stimuli, this non-invasive neural measure could provide a converging line of evidence for interpreting the genital and subjective arousal findings described above. In this study, we used fMRI to specifically focused on the “reward system” in order to address the question: to what extent is there an affective significance to findings from the literature on women’s sexual orientation and genital arousal?

The part of the “reward system” that we focused on is the ventral striatum, a dopamine-sensitive area of the brain that is a reliable measure of reward-related processing–and in particular, wanting and “incentive motivation”^28,29^–including with respect to sexual orientation^30^. Most neuroimaging studies of sexual response have focused on men^31,32^, but the ventral striatum has also been found to reliably activate in studies of women’s responses to erotic stimuli^33–35^. However, until now, no studies have measured neural responses to erotic stimuli in bisexual women.

The present investigation primarily focused on two hypotheses: (1) Homosexual women may show greater category-specificity than non-homosexual women in brain activity, as suggested by the genital arousal literature; (2) Bisexual women may show larger biases towards female stimuli, compared with heterosexual women. We tested these hypotheses with respect to subjective and neural responses to erotic pictures and erotic videos. We used two different kinds of erotic stimuli because of their potentially non-overlapping strengths and weaknesses. Erotic pictures may be particularly well-suited for assessing the initial appraisal of sexual stimuli, but their brevity may not reflect the kinds of experiences that drive sexuality in the real world. Erotic videos may allow for the measurement of more intense states, but their extended duration may also provide opportunities for self-regulatory efforts to modify erotic responses.

## Method

### Participants

Participants were 26 heterosexual women, 26 bisexual women, and 24 homosexual women, recruited from a variety of publicly-posted and online advertisements seeking (paid) volunteers for a neuroimaging study of sexual orientation and arousal. Bisexual women were required to have had at least two previous sexual partners and one romantic partner (of three months or greater duration) of each sex. Homosexual and heterosexual participants all met these criteria with respect to their respective preferred sexes.

After responding to advertisements, participants were screened for inclusion using online questionnaires. Participants provided information about sexual orientation, sexual interests, and personality, in addition to answering screening questions relevant to medical eligibility for fMRI research. Participants were required to be right handed, non-claustrophobic, free from ferromagnetic implants, and not currently taking psychiatric medications. Participants were informed of the risks and nature of the study and agreed to participate in all portions of the research. This study was approved by the Institutional Review Board of Northwestern University and carried out in accordance with its guidelines. Informed consent was obtained from each participant for every portion of the study in which they participated.

Participants’ sexual orientation was assessed using self-reported identities (i.e. “Homosexual”/“Gay”, “Bisexual”/“Bi”, “Heterosexual”/“Straight”), as well as with a modified Kinsey score, which asked participants about their sexual fantasies throughout adulthood as well as in the past year. The scale ranged from 0 to 6, with 0 corresponding to an exclusively heterosexual orientation and 6 corresponding to an exclusively homosexual orientation. Responses to questions about adulthood and about the past year were averaged to create a Kinsey score for each participant. The average Kinsey score was 0.8 for heterosexual women (*SD* = 0.7, *range* = 0–2), 2.63 for bisexual women (*SD* = 0.7, *range* = 2–4.5), and 5.2 for homosexual women (*SD* = 0.68, *range* = 4–6).

Participants’ ages ranged from 21 to 46 years old. Mean ages were 29.7 for heterosexual women (*SD* = 5.86, *range* = 25–46), 30.27 for bisexual women (*SD* = 6.41, *range* = 21–48), and 29 for homosexual women (*SD* = 3.12, *range* = 25–38). The sample of 76 participants was racially and ethnically diverse, with 23 non-Caucasian participants including two Latina participants, ten African-American participants, four Asian-American participants, and seven participants who identified otherwise or who identified as multiethnic/multiracial. Groups did not significantly differ either with respect to age (*F*(2,73) = 0.348, *p* = 0.708) or ethnicity (c^2^(2, N = 76) = 2.94, p = 0.23). We also confirmed that ethnicity did not significantly impact responses to the erotic stimuli.

### Stimuli and Procedure

Subjects experienced two experimental paradigms in the scanner: first erotic pictures were shown (over a period of ~21 minutes), and then erotic videos were shown (over a period of ~19 minutes) after a brief rest period. Picture stimuli were shown before video stimuli for all participants in an attempt to promote stimulus engagement. That is, it was assumed that potentially less intense stimuli might be better presented earlier in the experimental session while attentional resources are highest. Further, there was concern that first showing more intense stimuli would reduce engagement with subsequent stimuli. As such, pictures and videos stimuli were not counterbalanced with respect to each other.

Participants watched stimuli while laying down with a combination of earplugs (to minimize scanner noise) and over-ear headphones (for video sound and communication with experimenters). Images were displayed via projector onto a wall, which was made viewable to participants via an angled mirror placed above the eyes.

#### Erotic pictures paradigm

The present study employed a subset of the picture stimuli used in Safron *et al*.^36^ and Sylva *et al*.^7^. Pictures depicted a nude man, a nude woman, or a same-sex couple (i.e., either two men or two women) engaged in explicit sexual contact. Erotic stimuli featuring both individual nudes and same-sex pairs engaging in explicit sexual interaction is common in research on sexual arousal and sexual orientation^2,4,37^, which is not the case when stimuli featuring male-female couples is presented. However, erotic stimuli featuring explicit sexual activity in same-sex couples tends to be substantially more arousing compared with pictures of single nudes^4^. Such stimuli are similar to pictures of nude individuals, in the sense that only men or women, but not both, are depicted in a given picture. Thus, sexual arousal induced by them is relatively unambiguous in terms of the gender to which participants are responding.

In each of two 10.5-minute runs (ordering counterbalanced), participants viewed 40 erotic pictures featuring male models and 40 erotic pictures featuring female models. Each picture was shown for 3.5 seconds, followed by a variable-duration fixation cross presented for either 1.5, 6.5, or 11.5 seconds. Variable-duration baselines were utilized for superior deconvolution of the BOLD signal in a rapid event-related design for fMRI (in which evoked signals are never allowed to return to baseline levels). During the presentation of each picture, participants used buttons held in their right hands to rate that image on a scale of −2 to +2 (respectively: “strongly disliked,” “disliked,” “liked,” “strongly liked”), with no option of 0 for neutral ratings. Neutral options for ratings were not provided for the sake of consistency with previous research using the same stimuli. Note: Subjective ratings of pictures were lost for some participants due to a button-box equipment error.

#### Erotic videos paradigm

Following picture assessment, participants were shown six video clips depicting individual masturbating men and six video clips depicting individual masturbating women. Depicted individuals appeared sexually aroused but did not reach orgasm. To estimate baseline responses, six natural landscape videos were shown.

In each of two 9.25-minute runs (ordering counterbalanced), videos were presented for 15 seconds each, followed by a 15-second distraction task requiring participants to indicate via button-press when a number in a series decreased by an interval other than seven. This task was intended to facilitate a return to emotional and physiological baseline. 15-second stimulus presentations were chosen as a desirable stimulation period in an fMRI block design, which can potentially be more sensitive than event-related designs^38^.

After leaving the scanner, participants viewed the videos once more and provided ratings of each clip. Videos were rated using a 5-point scale for degree of sexual appeal, ranging from “not at all” (0) to “very much” (4), with a midpoint of “somewhat” (2).’

### fMRI signal extraction methods

#### Image acquisition

A Siemens Trio 3 T magnet and 12-channel RF head coil were used to collect T2*-weighted gradient-recalled EPI images from the whole brain (32 3-mm slices with a 0.99-mm interslice gap; TR = 2500 ms; TE = 20 ms; flip angle = 80°; FOV = 200 × 220 mm, 120 × 128 acquisition matrix). Slices were taken along the plane connecting the anterior and posterior commissures, with a 1.72 mm × 1.72 mm × 3.99 mm resolution, with more refined axial dimensions intended to produce less distortion and signal dropout in sub-cortical areas, although possibly at the expense of signal-to-noise ratio. During each picture run, 250 whole-brain volumes were collected, and during each video run, 220 whole-brain volumes were collected, with the first four volumes discarded to account for initial magnetization effects. For anatomical localization, a structural MRI scan consisting of T1-weighted images was conducted after the testing runs (160 1-mm axial slices; TR = 2.1 ms; TE = 4.38 ms; flip angle = 15°; FOV = 220 mm; 256 × 192 matrix).

#### Image pre-processing

Image pre-processing and analysis was performed using SPM 12b (Wellcome Trust Centre for Neuroimaging, London, UK), and implemented in Matlab v 8.1.604 (The MathWorks Inc., MA, USA).

Functional (EPI) volumes were first corrected for slice timing. Each participant’s volumes were then registered to the mean slice, after which the registered volumes were resliced, used to create a mean resliced image, and then co-registered to the structural (T1) image. All EPI images, including the mean resliced image, as well as the structural (T1) scans were then spatially normalized to Montreal Neurological Institute (MNI) space, and re-sampled to 3 × 3 × 3 mm (27 mm^3^) resolution. Normalized functional images were then smoothed to an 8 mm full-width-at-half-maximum Gaussian kernel.

#### Signal to noise ratio and head coverage exclusions

To exclude participants with poor signal due to either head motion or scanner conditions, average signal-to-noise ratio (SNR) over time was calculated for each subject (after preprocessing, using a mask that included only voxels with appreciable EPI signal). The SNR ratio for each voxel (mean divided by standard deviation) was averaged across all voxels in the brain^39^. Participants whose picture data SNR was less than one standard deviation below the mean were excluded from picture analyses. Similarly, participants whose video data SNR was less than one standard deviation below the mean were excluded from video analyses.

Based on these criteria, fourteen participants (five heterosexual, five bisexual, and four homosexual) were excluded from fMRI and subjective picture analyses, and sixteen participants (six heterosexual, six bisexual, and four homosexual) were excluded from fMRI and subjective video analyses. After exclusions were performed for SNR, we included a total of twenty-one heterosexual women, twenty-one bisexual women, and twenty homosexual women in fMRI picture analyses. Video analyses after SNR exclusion included eighteen heterosexual women, eighteen bisexual women, and twenty homosexual women. To check the validity of our SNR criterion, head motion plots were visually inspected for all participants (Parrish, *et al*.^39^). Excluded participants had highly variable head positions as compared to included participants. An additional validity-check was performed using evoked responses to erotic pictures minus a fixation-cross baseline. Excluded participants had substantially reduced activity in visual cortices as compared to included participants.

An additional thirty-two participants (twelve heterosexual, twelve bisexual, and eight homosexual) were excluded from subjective picture rating analyses due to insufficient subjective data resulting from a button-box equipment error. Five participants (three bisexual and two homosexual) were excluded from subjective video analyses for the same reason. Thus, after exclusions were performed for insufficient subjective data, we included a total of nine heterosexual women, nine bisexual women, and twelve homosexual women in subjective picture analyses, and twenty heterosexual women, seventeen bisexual women, and eighteen homosexual women in subjective video analyses.

For whole-brain analyses, mean functional scans were individually examined to identify participants with substantial cutoffs in head coverage. As a result, one heterosexual female who had substantial frontal lobe cutoff was excluded from whole-brain analyses in addition to those participants excluded for SNR.

#### First-level analyses

For both the video and picture assessments, a standard general linear model (GLM)^40^ was used in identifying hemodynamic changes for each participant, and a high-pass filter (cutoff 128 s) was used to remove low-frequency temporal noise.

Estimated average activity was calculated for each participant’s separate responses to male pictures, female pictures, male videos, and female videos (contrasted with fixation cross for pictures and neutral nature scenes for videos). These estimates were used for region of interest analyses. For whole-brain analyses, estimated average activity was also calculated for each participant’s response to male compared with female pictures and videos. For both the picture and video assessments, each participant’s responses to each stimulus contrast of interest were concatenated within stimulus type, using data from both the 1^st^ and 2^nd^ runs.

Ventral striatum region of interest analyses. An a priori region of interest (ROI) analysis was performed on the ventral striatum—centered on the nucleus accumbens—as this was the area most likely to indicate desire. The ventral striatum and hypothalamus are the only two areas that have been shown to be specifically associated with sexual (as opposed to general) arousal^41,42^. We focused on the ventral striatum because it likely has higher validity for reflecting sexual incentive value compared with the hypothalamus, which contains a variety of nuclei with heterogeneous functions (including sexual arousal) that would be difficult to disambiguate with the limited spatial resolution of 3 T fMRI.

**Figure 1:**
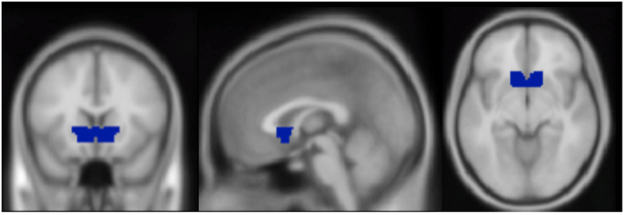
Mask used as the ventral striatum (VS) ROI, drawn using an average brain in the WFU PickAtlas toolbox for SPM 8. MNI coordinates displayed: x = 0, y = 17, z = −8.

The ventral striatum ROI mask used in the present study was drawn on an MNI template brain using the WFU PickAtlas toolbox for SPM 8^43^. It was anatomically defined as a dilated intersection of the ventral anterior caudate and putamen. The resulting ventral striatum ROI is shown in Fig. 1.

Estimates of average ventral striatum activity for each participant were extracted using the MarsBar toolbox for SPM8^44^. Extracted ventral striatum ROI data were analyzed using JMP Pro v11 (SAS Institute, Cary, NC).

### Planned contrasts and within-group tests

We constructed separate dependent variables for each combination of stimulus type (i.e. picture or video) and response type (i.e., subjective or ventral striatum activation) by subtracting response to female stimuli from response to male stimuli. That is, we constructed dependent variables for 1) subjective response to pictures, 2) subjective response to videos, 3) ventral striatum activation to pictures, and 4) ventral striatum responses to videos, each of which reflected responses to male stimuli minus responses to female stimuli. We refer to this as the Male-Female contrast.

Because there were three groups (i.e., heterosexual, homosexual, and bisexual women), two orthogonal between-groups contrasts were constructed to examine what we believe to be the most interesting pair of independent questions based on previous literature^45^. The first question was whether homosexual women differed from the other two groups in their Male-Female contrasts. The second question was whether bisexual women differed from heterosexual women in their Male-Female contrasts. The use of orthogonal planned contrasts allowed us to test these hypotheses with maximum statistical power while simultaneously minimizing the number of overall comparisons.

Within-group t-tests were also performed separately in each group in order to characterize relative responding to male and female stimuli.

### Whole-brain analyses

Finally, we examined overall patterns of differential activation in response to male compared with female erotic stimuli across the entire brain. If bisexual and heterosexual women have less specific arousal patterns, then they are likely to exhibit less extensive differential activity between male and female stimuli compared with the activity patterns expected for homosexual women.

Tests of average group responses to stimulus conditions were performed using one-sample contrasts. Each group (heterosexual women, bisexual women, and homosexual women) was tested individually for clusters of greater activity for male stimuli compared with female stimuli, and female stimuli compared with male stimuli, using a corrected statistical threshold (p < 0.05 FWE).

For these analyses, cluster reports were generated in SPM. Peak activations and cluster extents (extent threshold k = 5) were visually examined as overlays on slice and render maps. Neuroanatomical descriptions were determined based on agreement between two trained investigators, and checked against designations from the WFU Atlas (Maldjian *et al*., 2003).

### Data availability statement

The datasets generated and analyzed during the current study are available from the corresponding author on request.

## Results

### Between-group planned contrasts

As previously described, planned comparisons for the ventral striatum ROI were conducted via multiple regression using two orthogonal between-groups contrasts: one comparing homosexual women with heterosexual and bisexual women, and one comparing heterosexual with bisexual women. Separate analyses were conducted for each of the Male-Female contrasts (i.e., responses to female stimuli subtracted from responses to male stimuli). Results are presented in Table 1.

**Table 1:** Planned contrasts comparing women of different orientation groups.

	Variable: Male – Female		
	*β*	*t(df)*	*p*
**PICTURES**			
**Subjective Ratings**			
Homosexual vs. non-homosexual	−0.489	−2.60 (28)	0.015*
Bisexual vs. heterosexual	0.094	0.4 (28)	0.692
**Ventral Striatum Activation**			
Homosexual vs. non-homosexual	−0.462	−3.32 (59)	0.002**
Bisexual vs. heterosexual	−0.194	−1.22 (59)	0.226
**VIDEOS**			
**Subjective Ratings**			
Homosexual vs. non-homosexual	−0.820	−5.80 (55)	<0.001***
Bisexual vs. heterosexual	−0.373	−2.29 (55)	0.026*
**Ventral Striatum Activation**			
Homosexual vs. non-homosexual	−0.199	−1.38 (57)	0.172
Bisexual vs. heterosexual	−0.114	−0.688 (57)	0.494

*Significant p-value < 0.05. **Signifcant p-value < 0.01.

***Significant p-value < 0.001.

**Figure 2:**
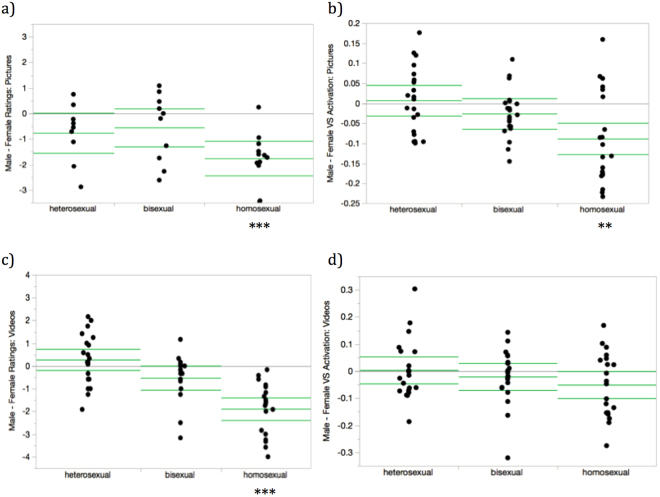
Within-group male – female (male minus female) stimuli difference scores for subjective ratings and ventral striatum (VS) responses, by sexual orientation. Difference scores are defined as a participant’s average response to stimuli depicting males minus average response to stimuli depicting females. Points represent individual participants. Horizontal bars indicate group means and 95% confidence intervals of the means. Horizontal lines at 0 indicate no difference between ratings to erotic stimuli depicting each sex. (**a**) Difference scores for subjective ratings of picture stimuli. (b) Difference scores for VS activation evoked by picture stimuli. (c) Difference scores for subjective ratings of video stimuli. (d) Difference scores for VS activation evoked by video stimuli. ***p < 0.001, **p < 0.01, *p < 0.05.

#### Homosexual versus non-homosexual women

Subjective ratings. Compared with non-homosexual women, homosexual women had significantly more negative (i.e., gynephilic) Male-Female contrasts for both pictures (p = 0.015) and videos (p < 0.001). That is, homosexual women showed a greater preference for pictures and videos of females relative to males, compared with both bisexual and heterosexual women.

Ventral striatum activation patterns. Homosexual women had significantly more female-biased ventral striatum responses compared to non-homosexual women for pictures (p = 0.002), but not videos.

#### Bisexual versus heterosexual women

We compared heterosexual and bisexual women’s subjective and ventral striatum responses to erotic pictures and videos, and observed only one significant difference: for video stimuli, bisexual women had significantly more female-preferring subjective responses than did heterosexual women (p = 0.026).

### Within-group tests comparing responses to male and female erotic stimuli

Figure 2 (showing the distribution of Male-Female contrasts for the three groups) shows that heterosexual women exhibited a non-significant trend (p = 0.079) towards favoring female erotic pictures compared with male erotic pictures, and had no differentiation between stimulus sex for other tests. Bisexual women also did not subjectively differentiate among stimulus types based on sex, although they did exhibit (non-significant) marginal female-favoring ventral striatum scores for picture (p = 0.063) and video (p = 0.054) stimuli. Homosexual women, in contrast to heterosexual and bisexual women, showed clear favoring of female stimuli as assessed by subjective liking of pictures (p < 0.001), appeal ratings of videos (p < 0.001), as well as in ventral striatum responses to pictures (p = 0.003) and non-significantly for ventral striatum responses to videos (p = 0.073). Note that these results are presented descriptively. Inferences about differences among the three groups depend on the tests presented in Table 1.

### Whole brain tests comparing responses to male and female erotic stimuli

Note: Activation patterns are described in greater detail in the discussion, with interpretations of possible functional significances.

#### Picture stimuli

**Figure 3:**
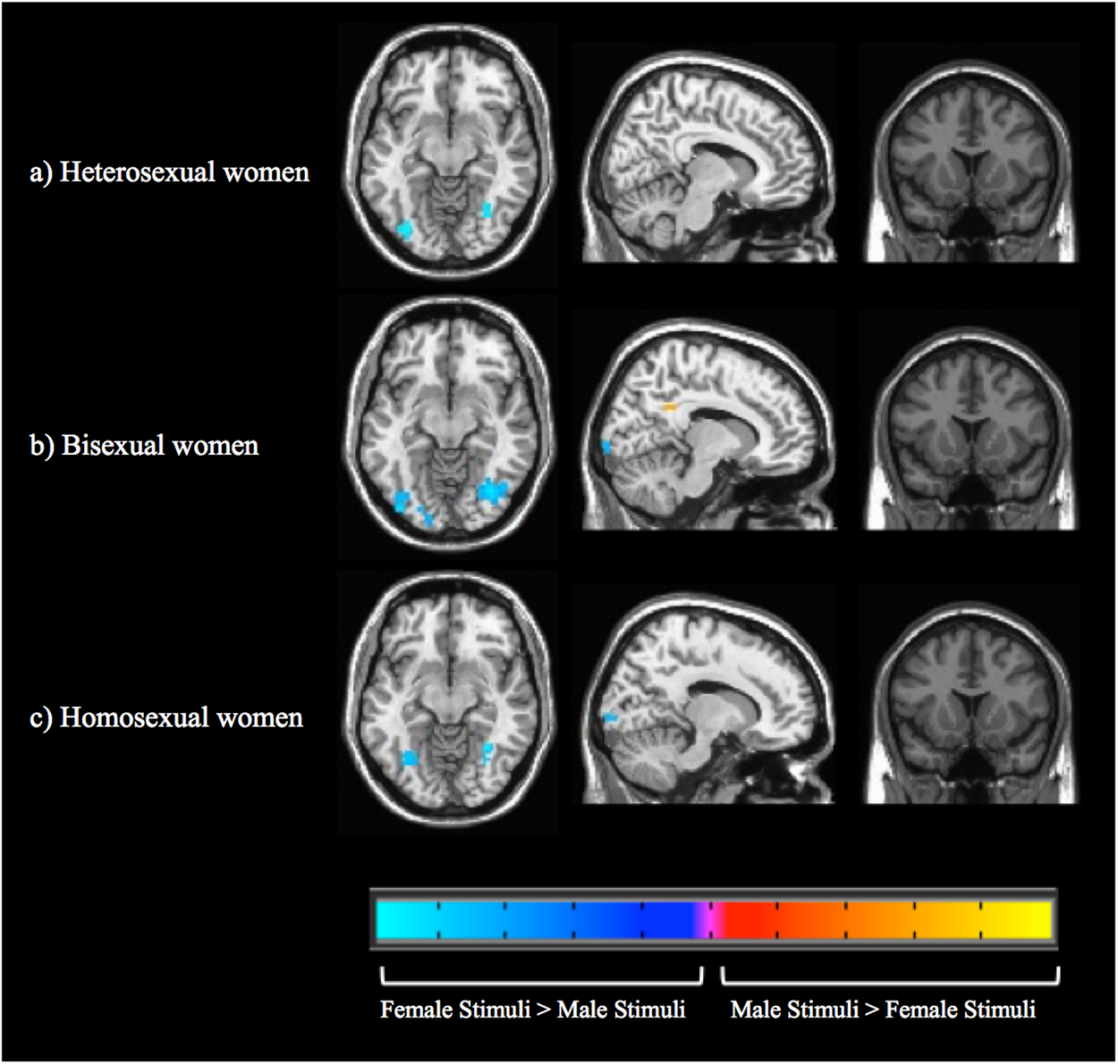
Differential brain activations towards male and female pictures in heterosexual, bisexual, and homosexual women. Whole brain activations are shown for the male picture minus female picture contrasts (with brain activation evoked by viewing neutral stimuli subtracted from activations toward the erotic pictures). Height threshold is set at p < 0.05 FWE with a cluster threshold of k = 5. Axial slice 31, sagittal slice 50, and coronal slice 38 are shown for all groups.

Comparing activation to female versus male erotic pictures, heterosexual women exhibited relatively greater activity for female pictures in occipital (i.e., visual) and occipitotemporal cortices, with no brain areas showing significantly greater activation for male pictures (Fig. 3; Table 2). Bisexual women also showed greater activity in visual cortices for female relative to male pictures, but they showed greater activity for male pictures in other areas including supramarginal and angular gyri, as well as the posterior cingulate. Homosexual women exhibited significant activations for female compared with male pictures in visual cortex, parietal lobes, and parahippocampal cortex, but with no brain areas showing significantly greater activation for male pictures.

**Table 2:** Differential whole-brain activations in response to male vs. female pictures.

R/L	Region	BA	MNI	voxels	peak T
*Heterosexual Women*					
Female > Male Pictures					
L	lingual gyrus, inferolateral occipital	18, 19	(−33 −76 −10)	21	8.01
R	fusiform gyrus	37	(30 −67 −7)	26	7.81
R	inferolateral occipital, fusiform gyrus	19, 37	(42 −70 −16)		7.78
R	fusiform gyrus	37	(30 −58 −10)		7.59
Male > Female Pictures: no differential activations					
*Bisexual Women*					
Female > Male Pictures					
R	inferior occipital cortex, middle occipital gyrus, lingual gyrus	18, 19	(36 −70 −10)	86	10.68
R	middle occipital gyrus	19	(48 −64 −10)		7.96
R	fusiform gyrus	37	(33 −55 −13)		7.15
L	inferolateral occipital, middle occipital gyrus	19, 18	(−39 −85 −7)	138	9.07
L	middle occipital gyrus	18, 19	(−30 −88 5)		8.47
L	inferior occipital gyrus, primary visual cortex, lingual gyrus	18, 17	(−24 −94 −4)		8.06
R	occipital cortex, middle occipital gyrus	19	(30 −79 17)	43	9.03
R	occipital cortex, middle occipital gyrus	19	(30 −82 26)		7.38
L	inferior occipital cortex, lingual gyrus	18	(−24 −85 −13)	5	8.22
R	primary visual cortex, cuneus	17	(15 −100 −4)	12	8.09
R	middle occipital gyrus	18	(42 −82 −1)	27	8.05
R	middle occipital gyrus	18	(27 −85 2)		7.62
R	middle occipital gyrus	18	(33 −91 8)		6.86
Male > Female Pictures					
L	angular gyrus, supramarginal gyrus	39, 40	(−51 −64 41)	21	9.37
R/L	posterior cingulate	23	(0 −22 35)	10	8.23
R	angular gyrus	39	(48 −61 35)	27	7.94
R	supramarginal gyrus	40	(51 −61 44)		7.75
R	retrosplenial cingulate	30	(15 −52 29)	6	7.68
R	supramarginal gyrus	40	(57 −49 35)	5	7.38
L	supramarginal gyrus	40	(−36 −61 44)	5	7.08
*Homosexual Women*					
Female > Male Pictures					
L	middle occipital gyrus	19	(−27 −82 14)	139	10.01
L	middle occipital gyrus	18, 19	(−33 −88 −1)		8.99
L	middle occipital gyrus, primary visual cortex	18, 17	(−18 −97 2)		8.57
R	middle occipital gyrus	19	(33 −79 14)	89	9.83
R	inferior lateral occipital cortex	19	(42 −85 2)		8.19
R	middle occipital gyrus	19	(33 −70 8)		7.78
R	fusiform gyrus, posterior paraphippocampal gyrus	37	(33 −52 −10)	36	9.56
R	fusiform gyrus, middle occipital gyrus	18	(33 −70 −13)		8.03
R	primary visual cortex	17	(18 −94 −1)	11	8.56
L	fusiform gyrus, lingual gyrus, posterior parahippocampal gyrus	37	(−33 −61 −7)	26	8.45
L	fusiform gyrus	37	(−39 −61 −13)		7.62
R	precuneus, occipitoparietal	7	(24 −73 41)	6	7.18
Male > Female Pictures: no differential activations					

#### Video stimuli

**Figure 4:**
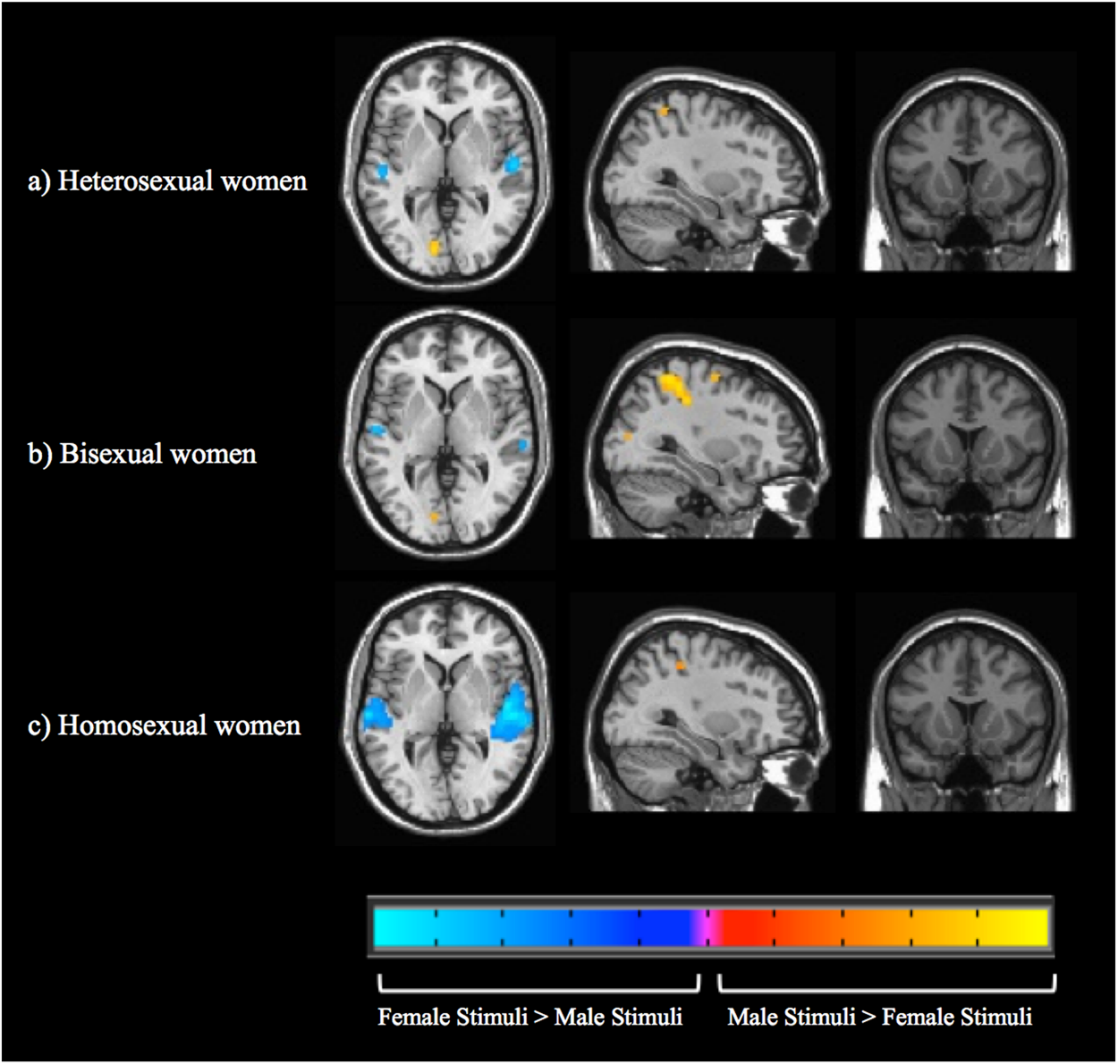
Differential brain activations between male and female videos in heterosexual, bisexual, and homosexual women. Whole brain activations are shown for the male video minus female video contrasts (with brain activation evoked by viewing neutral stimuli subtracted from activation toward the erotic videos). Height threshold is set at p < 0.05 FWE with a cluster threshold of k = 5. Axial slice 37, sagittal slice 61, and coronal slice 38 are shown for all groups.

When viewing female compared with male erotic videos (Fig. 4; Table 3), all groups showed activity in bilateral superior temporal cortices, likely indicating an auditory confound in which more extensive and substantial vocalizations were present in female erotic videos^46^. However, this effect appeared to vary by sexual orientation, with homosexual women showing the most extensive and robust evoked activity (peak T = 14.22) compared with heterosexual (peak T = 11.71) and bisexual women (peak T = 8.83). In the opposite direction of greater responses to male compared with female erotic videos, heterosexual and bisexual (but not homosexual) women exhibited activations in occipital cortices. While all groups had greater activity towards male videos in (anterior) superior parietal cortices, these activations appeared to be more extensive and robust in bisexual women (peak T = 11.55) compared with heterosexual (peak T = 7.64) and homosexual women (peak T = 7.99).

**Table 3:** Differential whole-brain activations in response to male vs. female videos.

R/L	Region	BA	MNI	voxels	peak T
*Heterosexual Women*					
Female > Male Videos					
L	superior temporal gyrus, primary and secondary auditory cortex	22, 41, 42	(−57 −25 8)	124	11.71
L	superior temporal gyrus, primary and secondary auditory cortex	22, 41, 42	(−42 −31 11)		10.15
L	superior temporal gyrus	22	(−66 −28 8)		8.99
R	superior temporal gyrus, primary and secondary auditory cortex	22, 41, 42	(54 −16 −1)	48	10.42
Male > Female Videos					
L	primary visual cortex, lingual gyrus	17, 18	(−9 −82 2)	30	10.14
R	inferolateral postcentral gyrus	3	(60 −16 29)	5	7.94
R	superior parietal lobule	5	(33 −49 62)	5	7.64
*Bisexual Women*					
Female > Male Videos					
L	superior temporal, primary and secondary auditory cortex	22, 41, 42	(−54 −10 5)	22	8.83
R	superior temporal gyrus	22	(63 −25 2)	8	7.26
Male > Female Videos					
R	superior parietal lobule, extending into supramarginal gyrus	7, 5, 40	(36 −46 59)	211	11.55
R	postcentral gyrus	3, 1, 2	(36 −37 53)		11.27
R	postcentral sulcus	1, 2	(33 −31 41)		9.51
L	postcentral gyrus, postcentral sulcus	3, 1, 2	(−36 −37 56)	25	8.45
L	superior parietal lobule	5, 7	(−33 −49 59)		7.55
R	supplementary motor area	6	(30 −10 59)	12	8.14
L	supplementary motor area	6	(−24 −13 62)	5	7.73
L	cuneus	17	(−9 −82 2)	9	7.6
*Homosexual Women*					
Female > Male Videos					
R	superior temporal gyrus	22	(51 −10 2)	578	14.22
R	primary and secondary auditory cortex, superior temporal gyrus	41 42, 22	(54 −19 5)		13.91
R	superior temporal gyrus	22	(60 −1 −4)		13.26
L	superior temporal gyrus	22	(−63 −19 2)	247	12.7
L	primary and secondary auditory cortex	41, 42	(−45 −25 8)		11.97
L	superior temporal gyrus	22	(−66 −28 5)		10.99
Male > Female Videos					
R	postcentral gyrus	3	(33 −34 47)	7	7.99
R	postcentral sulcus	2	(42 −28 41)	5	7.76

## Discussion

In this fMRI study of female sexual orientation—the first to include bisexual women—we extended several key findings from the sexual psychophysiology literature^1,2,47,48^. Using the ventral striatum as a neural measure of incentive motivation, we demonstrated that homosexual women have greater gender bias in their responses to male and female erotic stimuli.

### Main findings: subjective and ventral striatum responses to male and female erotic stimuli

Direct comparisons of bisexual and heterosexual women revealed no significant differences, with the exception of bisexual women having more gynephilic subjective responses to erotic videos. However, bisexual and heterosexual women did not differ with respect to their ventral striatum responses toward these stimuli. When contrasted to bisexual and heterosexual women, homosexual women showed distinctly greater bias toward female stimuli in both their subjective responses to videos and pictures, and also in their ventral striatum responses to pictures. In sum, our planned contrast findings are consistent with the genital arousal literature in which more category-specific responses were observed in homosexual women^2,4^.

Another set of tests, comparing male vs. female stimuli within each group, revealed that neither bisexual nor heterosexual women were significantly biased toward stimuli depicting males or stimuli depicting females. This was true both in ventral striatum response and in subjective arousal, for both picture and video stimuli. Homosexual women, however, were uniquely gynephilic (i.e., female-preferring), with significantly greater responses to female stimuli for subjective responses to pictures, subjective responses to videos, and ventral striatum responses to pictures. This gynephilic bias in homosexual women was consistent with our direct comparisons and previous literature.

Our findings are only partially consistent with observations from the genital arousal literature in which homosexual and bisexual women both had gynephilic responses, but where heterosexual women had non-specific responses^23–25^. We found significant biases in ventral striatum responses toward female stimuli among homosexual women, but with more indifferent patterns among heterosexual and bisexual women. However, with one exception—bisexual women showing more gynephilic subjective responses to erotic videos than did heterosexual women, in a direct comparison—heterosexual and bisexual women’s patterns of results did not differ significantly.

Our a priori tests in the ventral striatum allowed us to explore whether women of different sexual orientations also exhibited different degrees of incentive motivation toward male and female erotic stimuli. But fMRI also provides the ability to look at activation patterns across the entire brain, potentially allowing for a more detailed characterization of the neural systems involved. Below we review activation patterns for each group in viewing male compared with female erotic stimuli, along with some reverse inferences as to their functional significance.

### Whole brain responses to erotic pictures

For heterosexual women viewing erotic pictures, activity was greater for female relative to male stimuli bilaterally in lateral occipital cortices, likely indicative of visual attention^49,50^, as well as in right-lateralized fusiform cortex, potentially suggesting face or body processing^51,52^. *In no brain areas did heterosexual women have significantly greater activation for male relative to female erotic pictures*. Rather, they seemed to have a somewhat gynephilic pattern of visual attention, consistent with results from eye-tracking and looking-time studies in which heterosexual women attended to erotic characteristics of female pictures^53,54^.

Bisexual women showed more activity in response to female (relative to male) erotic pictures throughout the visual system, including fusiform cortex, which (as described above) is often associated with perception of faces and bodies^55^. Patterns were similar to those observed in heterosexual women (and presumably with similar functional significances), but with larger spatial extents of activation. Although bisexual and heterosexual women were not directly contrasted, this more extensive visual activation could be taken as support for somewhat greater gynephilic interest on the part of bisexual women, consistent with ventral striatum activation patterns.

For bisexual women viewing erotic pictures, activity was greater for male relative to female stimuli in posterior midcingulate and right retrosplenial cingulate cortices, potentially suggesting greater perceptual salience and emotional memory for male erotic stimuli^56–59^. Additional male-biased activations were identified bilaterally in supramarginal and angular gyri, indicating processes relating to mental imagery, or possibly mentalizing^60–64^.

Thus, in contrast to heterosexual participants, bisexual women showed greater activity towards male (relative to female) erotic pictures in affect-related brain areas. In this way, it seems that it would be overly simplistic to say that bisexual women are similar to heterosexual women, but with the addition of gynephilic interest. Rather, *bisexual women seem to have greater responses to both male and female erotic stimuli, depending on the brain area being considered*. Patterns of greater overall responsiveness are consistent with suggestions that bisexual women may be distinguished by having overall greater degrees of sexual motivation relative to heterosexual women^20,21^.

It is also notable that bisexual women uniquely showed greater activations to male stimuli in areas of the brain implicated in higher-order cognition, including mentalizing. Speculatively, these activations could be related to more complex processing of sexual motivation in bisexual women^65^. To the degree that these activation patterns in bisexual women actually specifically reflect social cognition, the question remains open as to why this may be more likely to be observed in bisexual but not heterosexual or homosexual women.

For homosexual women viewing erotic pictures, greater activations for female (relative to male) stimuli extended throughout the visual system, with additional clusters in occipitotemporal cortices. Clusters in the right inferior precuneus may have indicated mental imagery^66,67^, and clusters in posterior parahippocampal cortex may have indicated either memory encoding or retrieval^68,69^. *For homosexual women, no brain areas had significantly greater activation for male relative to female erotic pictures. Thus, homosexual women were the only group that exhibited an overall pattern of differential brain activity (between male and female sexual stimuli) greater only for pictures depicting their preferred gender*.

### Whole brain responses to erotic videos

For heterosexual women viewing erotic videos, activity was greater for female relative to male stimuli in bilateral superior temporal cortices likely indicating an auditory confound deriving from more extensive and substantial vocalizations being present in female erotic videos^46^. Activity was greater for male relative to female videos in posterior occipital cortex, likely indicating enhanced visual attention^70^. Further clusters (greater for male compared with female videos) in the inferolateral postcentral gyrus and parietal somatosensory association areas may have indicated awareness of bodily sensations, possibly related to sexual imagery^71,72^.

For bisexual women viewing erotic videos, activations were greater for female (relative to male) stimuli in superior temporal cortices, likely indicating the same auditory-related activity present in heterosexual women. Bisexual women’s brain activity was greater for male (relative to female) erotic videos in occipital cortex, likely indicating visual attention^49,51^. Male-biased activations in somatosensory cortices may have indicated processing of bodily sensations^73^, and further activations in bilateral superior parietal lobules, premotor and supplementary motor cortices, and right supramarginal gyrus may have indicated mental imagery or possibly mirroring with the actors shown in the videos^74–78^.

Similar to the findings for erotic pictures, *bisexual women were unique in the degree to which male videos produced activations in brain areas associated with more abstract (and possibly complex) processing*^79,80^. Again, the significance of this pattern remains unclear.

For homosexual women viewing female relative to male erotic videos, activity in superior temporal cortices likely indicated the same auditory-related processing observed in heterosexual and bisexual women, albeit more robustly and extensively, consistent with enhanced attention to emotionally salient stimulus features. When viewing male relative to female erotic videos, activations in the right somatosensory cortex may have indicated processing of bodily sensations^73^, which may have been either positive or negative in valence. Thus, while emotionally associated brain areas did not exhibit differential activations for videos, *homosexual women’s particularly strong engagement of auditory cortices for female stimuli provided yet further evidence of uniquely gender-biased responding, relative to heterosexual and bisexual women*.

### Comparisons with previous findings

Few studies have investigated the category-specificity of brain activity in non-heterosexual women. Ponseti *et al*.^81^ found that both heterosexual and homosexual women showed gender-specific patterns of brain activity in multiple areas, including the ventral striatum. Sylva *et al*.^7^ also found some evidence for category-specific responding in women, although not in the ventral striatum, and without specifically testing whether or not heterosexual or homosexual women differed in their responses.

The patterns observed Ponseti *et al*.^81^ stand in contrast to the present investigation in which homosexual women tended to be the only group showing strongly category-specific responses to erotic stimuli. One possible interpretations for their findings of category-specific responses in all women was the unusual nature of the stimuli (i.e., close-up images of male and female genitalia, isolated from interpersonal contextual factors)^82^. As suggested by Chivers (2017)^48^, it may be the case that sex and gender cues can produce specific responses in heterosexual women, but that these are usually trumped by contextual factors in driving arousal responses in women. The stimuli utilized in the present study contained contextual factors (e.g. body posture, facial expression) that are more typical of those found in the genital arousal literature.

However, it should be noted that the present study did not find support for greater category-specificity in homosexual women across all stimulus conditions. Rather, planned contrasts in the ventral striatum only revealed significant group differences between homosexual and non-homosexual women for erotic pictures. There were no significant differences in ventral striatum response between homosexual and non-homosexual women for erotic videos (even though subjective evaluations of those stimuli did significantly differ across the groups).

This pattern of differing results for pictures versus videos may be related to differences in how individuals respond to these stimuli, limitations of our video paradigm, or both. While erotic videos may theoretically allow for the assessment of qualitatively different states of sexual response, it may be the case that incentive motivation is greatest when stimuli are first presented, but then diminishes with longer stimulus presentations^83^. Additionally, erotic pictures may have been more effective at driving ventral striatum responses due to factors such as unpredictably varied presentation times of preferred stimuli contributing to larger magnitude reward-prediction errors^84,85^.

### Limitations

One limitation of nearly all studies of erotic responses in women—including this one—is a failure to control for hormonal conditions or contraceptive usage. By default, it can generally be assumed that most women were not measured within the ovulatory window, when responses to erotic stimuli might be greatest^35,86^. Additionally, a number of women may have been using hormonal contraceptives. Measuring women’s responses outside of the fertile phase of their cycles—or while they were using hormonal contraceptives^87^—may have yielded a restricted range of arousal responses. However, the specificity of erotic responding has not been shown to be influenced by menstrual cycle in previous studies of genital arousal^48^.

Another source of potential limitations may have been the nature of the stimuli used. Though our stimuli were pilot-tested and rated by individuals of different sexual orientations in order to confirm that they would appeal to a broad participant sample, it is never possible to ensure that common stimuli will evoke the responses intended. This may be especially true for something as emotionally salient and individual as sexual arousal. Thus, it is possible that category specificity patterns could appear to be different if stimuli better reflected participants’ subjective preferences. This is a limitation of many studies of sexual responding, although data gleaned from more individualized stimulus sets are difficult to interpret.

One more aspect of the stimuli that is difficult to control for is sensory details that are inherently different between male and female stimuli. Differences in actors’ vocalizations (for videos), actors’ body positions (for both videos and pictures), and actors’ body motions (in the videos) were present (on average) between male and female stimuli. These features are difficult to control for and could conceivably lead to differences in both subjective and neural responding when viewing male vs. female stimuli, especially in more primary sensory areas of the brain such as visual and auditory cortices. However, such differences may also serve to reinforce the gendered nature of the stimuli and improve their correspondence with real-world experiences and real-world arousal.

## Conclusions

Though the neural data presented here align with previously-observed patterns in women’s genital and subjective arousal, much remains unknown about the relationship between arousal patterns, orientation, and the development of sexual motivation towards particular sexes in women. Our study supports past findings indicating that women tend not to have strongly category-specific responses to erotic stimuli, with homosexual women showing somewhat greater specificity than heterosexual and bisexual women. Future research should explore the extent to which women’s non-specific sexual response contributes to erotic plasticity (i.e., change with context) and sexual fluidity (i.e., change over time)^9,10,88^.
